# Extracellular vesicles: immunomodulation, diagnosis, and promising therapeutic roles for rheumatoid arthritis

**DOI:** 10.3389/fimmu.2024.1499929

**Published:** 2024-11-18

**Authors:** Desalegn Abebaw, Yibeltal Akelew, Adane Adugna, Zigale Hibstu Teffera, Bantayehu Addis Tegegne, Abebe Fenta, Bantegize Selabat, Gashaw Azanaw Amare, Mamaru Getinet, Mohammed Jemal, Temesgen Baylie, Aytenew Atnaf

**Affiliations:** ^1^ Department of Medical Laboratory Science, College of Medicine and Health Sciences, Debre Markos University, Debre Markos, Ethiopia; ^2^ Department of Medicine, Centre for Inflammatory Diseases, Monash University, Clayton, VIC, Australia; ^3^ Department of Pharmacy, College of Medicine and Health Sciences, Debre Markos University, Debre Markos, Ethiopia; ^4^ Department of Biomedical Sciences, School of Medicine, Debre Markos University, Debre Markos, Ethiopia

**Keywords:** extracellular vesicles, immunomodulation, rheumatoid arthritis, therapeutic roles, diagnostic markers

## Abstract

Extracellular vesicles (EV) can be produced as part of pathology and physiology with increased amounts in pathological conditions. EVs can carry and transfer cargo such as proteins, nucleic acids, and lipids to target cells and mediate intercellular communication resulting in modulation of gene expression, signaling pathways, and phenotype of recipient cells. EVs greatly influence the extracellular environment and the immune response. Their immunomodulatory properties are crucial in rheumatoid arthritis (RA), a condition marked by dysregulated immune response. EVs can modulate the functions of innate and adaptive immune cells in RA pathogenesis. Differentially expressed EV-associated molecules in RA, such as microRNAs (miRNAs), long-noncoding RNAs (lncRNAs), messenger RNAs (mRNAs) and proteins are promising markers to diagnose the disease. miRNA, lncRNA, and circular RNA (circRNA) cargos in EV regulate inflammation and the pathogenic functions of RA fibroblast-like synoviocytes (RA-FLS). Downregulated molecules in RA tissue and drugs can be encapsulated in EVs for RA therapy. This review provides an updated overview of EVs’ immunomodulatory, diagnostic, and therapeutic roles, particularly emphasizing mesenchymal stem cell-derived EVs (MSC-EVs).

## 1 Introduction

Extracellular vesicles (EVs) are membrane-enclosed particles released by eukaryotic and prokaryotic cells as part of physiological and pathological processes with increased release under pathological conditions (1–3). EVs can carry various biomolecules, including proteins, nucleic acids, and lipids to the extracellular environment facilitating the transfer of their cargo to the recipient cells (4, 5). Recent evidence suggests that EVs may also contain mitochondria which control the epigenetics of target cells and organs (6).

The lipid bilayer of EVs encloses and protects their contents from the external environment (7). EVs vary in size from nanoscale exosomes to larger microvesicles and can be secreted by virtually all cell types (8). They are present in various body fluids including cerebrospinal fluid (CSF) (9), breast milk (10), synovial fluid (SF) (11), saliva (12), urine (13) and blood (14). Initially, EVs were considered mere cellular waste, leading to their limited investigation until recent years (15).

Traditionally, EVs were classified based on particle size and biogenesis into exosomes, microvesicles, and apoptotic bodies (4, 16). Exosomes, the smallest EVs, are formed through the inward budding of the plasma membrane during endosome generation, which matures into multivesicular bodies (MVBs) or late endosomes (7, 17). These MVBs either fuse with cell membrane to release exosomes or merge with lysosomes for degradation (1, 18). Microvesicles, (also called ectosomes) are generated through the outward budding and fission of the plasma membrane (19, 20), while apoptotic bodies, the largest EVs, are produced during programmed cell death and contain both cytoplasmic and nuclear materials (21, 22). However, no definitive molecular markers exist to distinguish these categories (8).

Given the limitations of biogenesis-based classification, the International Society for EVs recommended avoiding this terminology unless universal molecular markers and effective separation techniques are available. In its 2023 position paper, “Minimal Information for Studies of EVs (MISEV 2023),” the Society advocated using the general term “extracellular vesicles” and proposed size-based nomenclature, such as “small EVs (sEV)” for particles smaller than 200 nm and “large EVs (LEV)” for those larger than 200 nm (8).

EVs significantly impact the extracellular environment and immune responses (23, 24). They facilitate intercellular communication by transferring functional components or inducing receptor-mediated signaling (25). The surface proteins of EVs and their cargo can modulate gene expression, signaling pathways, and the phenotypes of target cells (15). EVs, produced endogenously, have advantages over synthetic nanoparticles and viral vectors, including higher biocompatibility, lower immunogenicity, and better evasion of phagocytosis (26). Moreover, their ability to cross biological barriers, such as the placental, blood-brain, blood-tumor, and blood-testis barriers, makes EVs promising tools for drug delivery (15, 27, 28). EVs have shown significant potential in the detection and treatment of autoimmune diseases including rheumatoid arthritis (RA) (29), multiple sclerosis (30), and type 1 diabetes (31).

RA is among the most prevalent chronic inflammatory disorders, affecting approximately 0.5% of the population globally (32, 33). This long-lasting autoimmune disease leads to the progressive destruction of joints. Despite advancements with disease-modifying antirheumatic drugs (DMARDs), treatment remains inconsistent, with 30-40% of patients discontinuing DMARDs due to ineffectiveness or side effects (34–36). Blocking tumor necrosis factor-α (TNF-α) helps reduce joint inflammation, prevent structural damage, and enhance the quality of life in 60-70% of RA patients. However, since some individuals don not respond to this therapy, alternative treatment options are necessary (37). Given the limitations of current treatments, researchers are increasingly exploring biotherapies, including EVs (38). In this review, we discuss what is presently known about the roles of EVs in RA’s immunomodulation, diagnosis, and therapeutic potential, with a special emphasis on mesenchymal stem cell (MSC)-derived EVs.

## 2 EV-induced immunomodulation in RA

Various immune cells, including T cells (39), B cells (40), macrophages (41), and mast cells (42) play a role in the progression of RA, with macrophages and T cell subsets playing particularly significant roles (43–45). T helper 1 (Th1) cells stimulate the production of interferon-γ (IFN-γ), TNF-α, and interleukin 2 (IL-2), contributing to cartilage damage and bone erosion, while, Th17 cells release IL-22, promoting the growth of synovial fibroblasts (46). B cells generate autoantibodies and drive autoimmune responses through the production of rheumatoid factor (40). Macrophages provide proinflammatory cytokines such as TNF- α and IL-1β (47).

New RA therapies have been proposed that focus on regulating the local immune response and promoting antigen-specific immune tolerance (48). EVs derived from MSC, neutrophils, granulocytic myeloid-derived suppressor cells (G-MDSCs), Dendritic cells (DC), and macrophages modulate the immune response within the inflammatory microenvironment of injured cartilage (49).

The type and condition of the source cell determine the influence of EVs on the immune response (50). MSC-derived EVs possess strong immunomodulatory properties, and their effectiveness is linked to their uptake by immune cells (51, 52). These EVs can regulate both innate and adaptive immune functions, reducing abnormal inflammation while ensuring safety in the surrounding microenvironment (53–55). This makes them a promising option for treating inflammatory diseases (56).

### 2.1 EV-induced innate immune modulation in RA

EVs influence the functions of innate immune cells, impacting processes like differentiation, activation, migration, and cytokine production, as well as their abilities in cytolysis, phagocytosis, and antigen transfer (57). Macrophages are crucial innate immune cells involved in the pathogenesis of RA (44, 58). In RA patients, there is an increase in pro-inflammatory M1 macrophages and a reduction in anti-inflammatory M2 macrophages (44). M1 macrophages secrete pro-inflammatory substances whereas M2 macrophages release anti-inflammatory agents (59). EVs can influence macrophage function by transferring regulatory miRNAs and proteins modulating inflammatory responses by affecting toll-like receptor 4 (TLR4) signaling and cytokine production (60, 61). M2 macrophage-derived EVs transfer proteins which can polarize macrophage to M2 phenotypes (62). Additionally, neutrophil-derived microvesicles can boost anti-inflammatory factors like transforming growth factor-β (TGF-β) and prevent inflammatory activation of synoviocytes in arthritis models (63).

MSC-derived EVs have been shown to promote M2 macrophages while reducing pro-inflammatory M1 macrophages in the synovial tissue of mice with collagen-induced arthritis (CIA) (64). Additionally, bone marrow MSC-derived EVs (BMSC-EVs) were found to inhibit the secretion of inflammatory cytokines including IL-1β, TNF-α, and IL-18 in macrophages from mice with RA (65). *In vitro* studies indicated that MSC-EVs can prevent DC maturation by downregulating the expression of CD80, CD83, and CD38, decreasing IL-6 and IL-12p70 secretion, and increasing TGF-β production. These findings suggest that MSC-EVs could be a promising therapeutic approach in mitigating autoimmune diseases such as RA by modulating dendritic cell function (66).

### 2.2 EV-induced adaptive immune modulation in RA

In RA, dysregulated immune responses activate auto-reactive T and B cells, leading to their proliferation and differentiation into pathogenic cells that produce autoantibodies, thereby driving joint inflammation and degradation (67).

**Figure 1:**
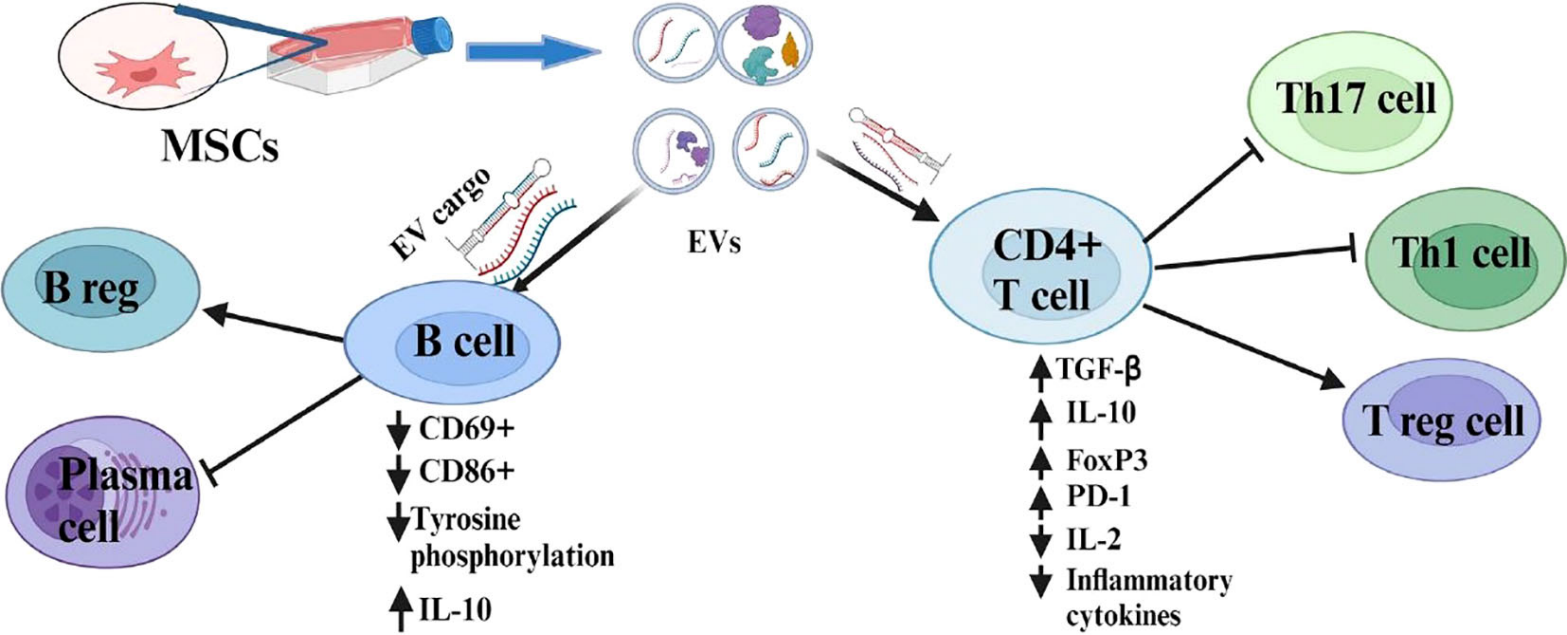
Immunosuppressive role of MSC-EVs and their modulatory effects on the adaptive immune cells.

EVs isolated from plasma of RA patients suppress early B cell activation in RA by downregulating the expression of activation markers like CD69^+^ and CD86^+^, and by inhibiting intracellular signaling pathways that are essential for B cell proliferation, function, and survival results (68). BMSCs and G-MDSCs release EVs that regulate B cell differentiation by promoting CD19^+^IL-10^+^ regulatory B (B reg) cells and reducing plasmablast phenotypes in the lymph node of mice with CIA (69, 70) (**Figure 1**).

Human gingival mesenchymal stem cell-derived EVs (GMSC-EVs) regulate CD4^+^ T cell subpopulations by increasing regulatory T (T reg) cells and decreasing Th1 and Th17 cells in the CIA model. Experiments conducted both *in vivo* and *in vitro* demonstrated that GMSC-EVs induce upregulation of anti-inflammatory cytokine (IL-10) and downregulation of proinflammatory cytokines including IFN-γ, IL-17A, TNF-α, and IL-6 (38). These EVs carry miR-148a-3p, which is responsible for immunomodulatory effects by directly targeting IKKB (inhibitor of nuclear factor kappa B kinase) in T cells (71). G-MDSC-derived EVs demonstrated a similar immunosuppressive effect on CD4 T cells in CIA mice. G-MDSC-EV cargos (miR-29a-3p and miR-93-5p) suppress the differentiation of Th1 and Th2 cells by targeting T-bet and signal transducer and activator of transcription 3 (STAT3), correspondingly (72).

EVs derived from human umbilical cord stem cells (hUCMSC-EVs) suppress T lymphocyte proliferation and induce apoptosis along with upregulation of forkhead box p3 (FoxP3) and downregulation of retinoic-related orphan receptor (RORγt) in the spleen of CIA mice (73, 74). These types of EVs demonstrated a Th1/Th17 and T reg cell balance accompanied by reduced levels of IL-17 and enhanced TGF-β and IL-10 in CIA mice (73–75). MSC-EVs derived from adipose tissue also modulate activated T cells by down-regulating miR23a-3p, which post-transcriptionally regulates TGF-β receptor 2 (*TGFBR2*) and increases the expression of FoxP3 (76). Furthermore, MSC-EV regulates the proliferation of activated T cells by inducing cell cycle arrest via upregulation of P27kip1 expression and downregulation of cdk2 expression (77). On the other hand, both CD4 and CD8 T cell proliferation were not affected in the presence of EVs derived from bone marrow MSC. However, an indirect inhibitory effect was observed through T reg cell induction resulting in a reduction of CD4 and CD8 T cells (69).

Research suggested that manipulating MSC-EVs could enhance a balance among Th cells and reduce the production of proinflammatory cytokines. EVs isolated from miR-146a transduced MSC resulted in upregulating FoxP3, TGFβ, and IL-10 and downregulating IFN-γ in CIA models (78, 79). More importantly, pro-inflammatory cytokine-priming of MSC-EVs does not affect its immunosuppressive potential (69, 80, 81). Moreover, EVs derived from interferon-β (IFN-β)-primed MSCs down-regulated the expression of RA-associated cytokines (IL-4, GM-CSF, IFN-γ, IL-2, TNF-α) and diminished CD4^+^ T-cell polyfunctionality in RA CD4^+^ T cells (82). Immortalized adipose tissue-derived MSCs primed with serum from RA disease conditions generate EVs that boost TGF-β1 production, promote Th2 induction, and facilitate M2 polarization, reducing inflammatory cytokines in CIA mice (83).

Under normoxic conditions (21% O2, 5% CO2), MSC-derived EVs promoted T reg cell phenotypes and reduced CD4+ T cell polarization toward Th17 phenotypes, demonstrating their immunomodulatory effects in an antigen-induced arthritis model (81). Under hypoxic conditions, EVs derived from polymorphonuclear myeloid-derived suppressor cells (PMN-MDSCs) suppress the proliferation of CD4+ T cells in the CIA mouse model (84). In contrast, synovial fibroblast-derived EVs in a hypoxic environment, reduce T reg cells and promote polarization of Th17 cells. Elevated levels of miR-424 under this condition downregulate FoxP3, thereby worsening RA (85).

EVs generated from TGF-β primed T reg cells effectively mitigated the Th17 and Treg cells imbalance in arthritic mice and regulated the inflammatory responses of recipient T cells via miR-449a-5p-dependent mechanism (86). Microvesicle mimetics (MVM) isolated from endotoxin-tolerant DCs possessed a bioactive miR155-3p and exhibited remarkable immunosuppression by inducing T reg and anti-inflammatory macrophages in RA models (87). In addition, in the RA microenvironment, EVs containing programmed death receptor 1 (PD-1) facilitate T cell exhaustion in the joints (88). Moreover, in RA patients, SF EVs expressing gangliosides (GD3), were associated with immunosuppression by inhibiting T cell activation after stimulation via TCR. This suggests that immunosuppressive EVs in the synovial fluid serve as a novel immune checkpoint for T cells (89).

## 3 EVs as a diagnostic marker of RA

The potential role of EVs in discovering specific biomarkers to diagnose various autoimmune diseases has been highlighted (2). The quantity of EVs is notably higher in the plasma and synovial fluid of individuals with RA than in healthy controls (90). EVs are recognized for containing distinct proteins that reflect the characteristics of their originating cells (91). Differentially expressed miRNA and lncRNAs containing EVs are also associated with RA’s immune response and metabolic process (92). By comprehending the variety of their contents and associated targets, it could be feasible to diagnose RA and other autoimmune diseases (93).

### 3.1 EV microRNAs (EV-miRNA) as a diagnostic marker of RA

miRNAs are short non-coding RNAs that play a role in cell signaling, intracellular communication, regulation of gene expression, and chronic inflammation and immune responses (94, 95). They are key regulators of skeletal remodeling and play a role in the development of RA (96). SF from joints exhibiting high-grade inflammation had 3.5 times more miRNA-positive EVs per ml than normal levels. Analysis of the most prevalent miRNAs indicated that they negatively regulate several inflammation-related genes, including STAT3, which play a pro-inflammatory role in RA (97).

While various EV-miRNAs have been investigated for HBV-related tumor detection (98), several studies have shown promising results in using EV-associated miRNAs for RA diagnosis (**Table 1**). Dysregulated RNAs in sEVs derived from FLS associated with arthritis in mice models were highlighted as a potential biomarker for RA (99). The miRNA content of EVs, such as miR-212-3p, miR-338-5p, miR-410-3p, and miR-537, showed elevated levels in early RA during methotrexate (MTX) treatment, suggesting their potential as diagnostic and prognostic biomarkers (100). miRNA cargos such as (hsa-miR-335-5p and hsa-miR-486-5p) were higher in the peripheral blood of RA patients than in healthy controls and associated with disease activity (101). Furthermore, miRNA-1915-3p containing EVs were elevated in the clinical remission group of Korean RA and negatively correlated with serum C-reactive proteins (CRP) levels and may be useful to indicate RA disease activity (102).

**Table 1:** Expression of various miRNA, lncRNA, mRNA, and proteins in EV during RA.

EV associated biomarker	Biomolecule	EV source	Detection method	Expression level	Ref.
**miR-204-5p**	miRNA	plasma	qRT-PCR	downregulated	(127)
**DPYSL3**	protein	CD4^+^T cells	Proteomics	upregulated	(114)
**PSME1**		CD4^+^T cells	Proteomics	downregulated	
**miR-221**	miRNA	synovial fluid	qRT-PCR	upregulated	(99)
**miR-45a & miR-25-3p**	miRNA	serum	qRT-PCR	upregulated	(105)
**hsa-miR-335-5p**	miRNA	blood	qRT-PCR	upregulated	(101)
**hsa-miR-486-5p**					
**TCONS_I2_00013502**	lncRNA	serum	qRT-PCR	upregulated	(109)
**ENST00000363624**	lncRNA	serum	qRT-PCR	downregulated	
**NONHSAT193357.1**	lncRNA	serum	qRT- PCR	downregulated	(92)
**CCL5**	mRNA	serum	qRT- PCR	downregulated	
**MPIG6B**	mRNA	serum	qRT- PCR	downregulated	
**IgM**	protein	Plasma	ELISA	upregulated	(115)
**SNHG6**	lncRNA	plasma	qRT-PCR	upregulated	(110)
**RPS18P9**	lncRNA	plasma	qRT-PCR	upregulated	
***CXXC4-AS1***	lncRNA	plasma	qRT-PCR	downregulated	
**ENST00000433825.1**	lncRNA	synovial fluid	qRT-PCR	upregulated	(107)
**miR-6089**	miRNA	serum	qRT-PCR	downregulated	(61)
**miR-144-3p**	miRNA	plasma	qRT-PCR	downregulated	(128)
**miR-30b-5p**	miRNA	plasma	qRT-PCR	downregulated	
**miR-885-5p**	miRNA	serum	qRT-PCR	upregulated	(129)

CCL5; chemokine c-c motif ligand 5, DPYSL3; dihydropyrimidinase-related protein 3, IgM; Immunoglobulin M, lncRNA; long noncoding RNA, MPIG6B; megakaryocyte and platelet inhibitory Receptor G6b, miRNA; microRNA, PSME1; proteasome activator complex subunit 1, qRT-PCR; quantitative real-time PCR, RPS18P9; ribosomal protein s18 pseudogene 9, SNHG6; small nucleolar RNA host gene 6.

The bold text indicates Rheumatoid arthritis (RA) biomarkers associated with extracellular vesicles (EVs).

Differentially expressed miRNAs linked to RA pathogenesis, such as miR-155-5p, miR-146a-5p, miR-323a-5p, and miR-1307-3p, were found in EVs derived from RA synovial fibroblast cell lines after TNF-α stimulation (103). Based on the serum EV expression profiles, patients with RA exhibited elevated levels of variably expressed miR-125a-5p, miR-130b-3p, miR-151a-5p, miR-301a-3p, and miR-324-5p (104). A combination of sEV miRNAs and soluble tumor necrosis factor-like weak inducer of apoptosis (sTWEAK) diagnosed early RA with a sensitivity of 85.7% and a specificity of 100% (105) (**Table 1**).

### 3.2 EV-Long noncoding RNAs (EV-lncRNA) as a diagnostic marker of RA

Long non-coding RNAs (lncRNAs) represent a new category of non-coding RNAs that do not produce proteins (106). The expression profiling of lncRNAs in EVs obtained from the synovial fluid of RA demonstrated significant differences when compared to osteoarthritis (OA) and gout (107). The serum sEV lncRNA profiles in patients with RA were also distinct from those of healthy controls and patients with OA (92).

The expression of circular RNAs (circRNAs), such as circFTO, is elevated in EVs derived directly from FLS of RA patients. These EVs promote RA progression by suppressing chondrocyte growth and migration while enhancing apoptosis and catabolism (108). Variably expressed lncRNAs in serum EVs from RA patients showed both upregulation and downregulation (109). lncRNAs found in plasma EVs from individuals with RA exhibit distinct expression profiles, including several lncRNAs that may serve as diagnostic biomarkers. The receiver operating characteristics curve (ROC), which is used to evaluate the diagnostic accuracy of biomarkers, revealed that lncRNAs including *SNHG6, RPS18P9*, and *CXXC4-AS1* demonstrated an area under the curve (AUC) ranges of 0.847-0.994 in diagnosing RA (110).

### 3.3 EV-associated protein and mRNAs as diagnostic markers of RA

Analysis of differentially expressed proteins in EVs from SF revealed that stromelysin-1 and pregnancy zone protein (PZP) were among the highly expressed proteins in RA as compared to OA (111). Proteomic analysis found that EVs from RA-FLS had higher pentraxin (PTX3) and lower proteasome 20S subunit beta 5 (PSMB5) levels than OA patients, promoting macrophage migration and RA progression (112). Lipid binding protein (LBP) and monocyte differentiation antigen (CD14) were also upregulated in EVs. Notably, the interaction of these proteins may play a role in nuclear factor kappa B (NF-κB) signaling, promoting the expression of IL-8 and TNF-α, which could contribute to the development of RA and serve as potential biomarkers for its diagnosis (113).

Differentially expressed proteins were identified as both upregulated and downregulated in the CD4^+^ T cell-derived EVs of RA patients, suggesting that these proteins could act as potential biomarkers for RA (114). The levels of CD3**^+^** CD4**^+^** protein containing EVs in the serum of RA patients are elevated, whereas the levels of CD3^+^CD8^+^ EVs are reduced, reflecting that total CD4^+^ T cells are dominant over CD8^+^ T cells (91).

In a subset of seropositive RA patients, rheumatoid factor immunoglobulin M (IgM-RF) was found on plasma EVs and associated with increased disease activity. This discovery suggests a potential biological factor that could explain the discrepancy between global disease activity assessments and the counts of tender and swollen joints (115). Elevated levels of circulating EVs testing positive for immunoglobulin G (IgG), IgM, CD41a, and citrulline were also observed in seropositive RA patients (116). Profiling of plasma EVs identifies proteins significantly linked to the patient’s global disease activity (PGA) in RA. Notably, actin-cytoskeleton linker proteins, including ezrin and moesin, correlate positively with PGA (117).

**Figure 2:**
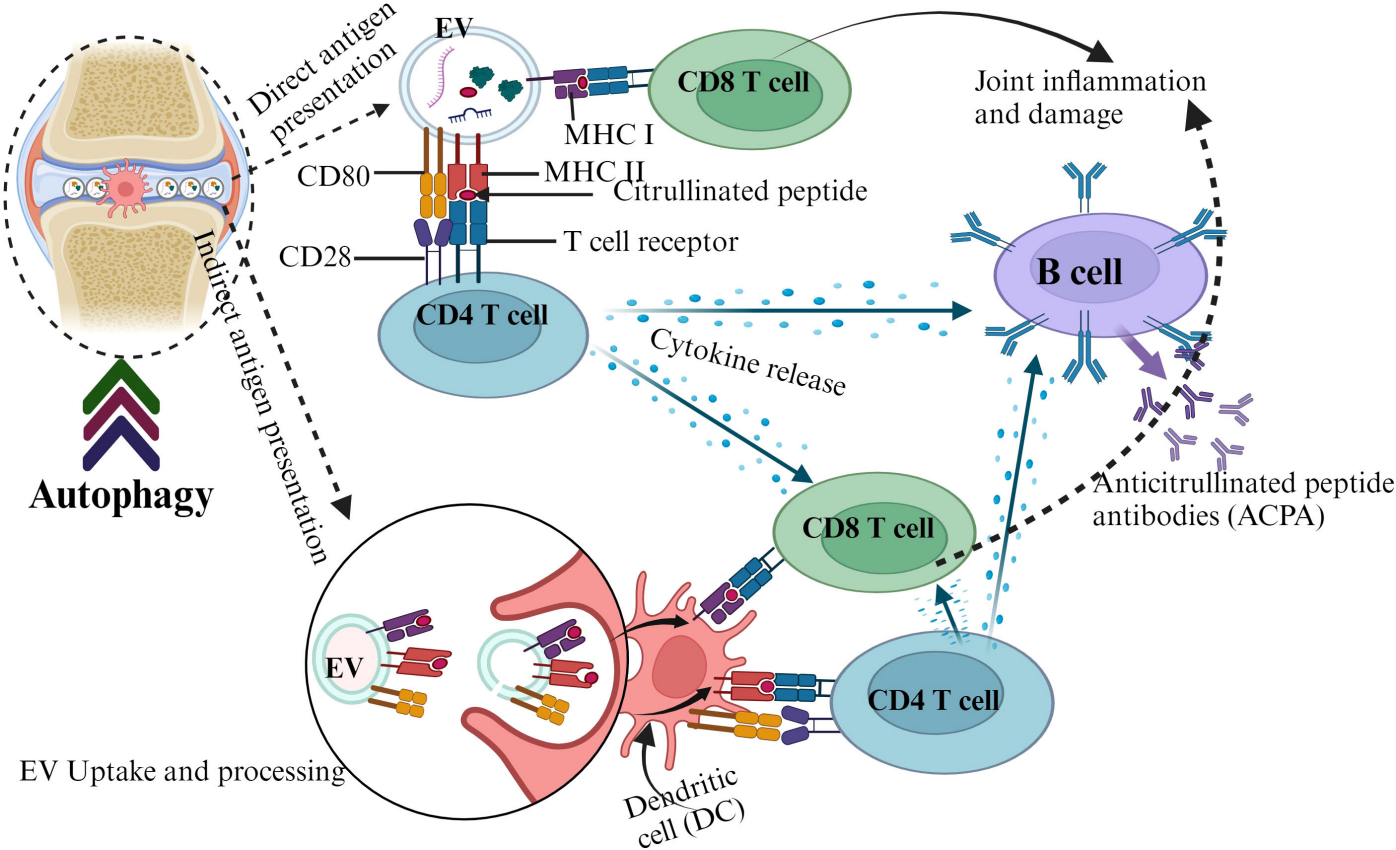
The interplay between autophagy, EVs, and autoantigen presentation in RA.

Circulating EVs express elevated levels of posttranslational modified proteins such as citrullinated proteins and contribute to the pathogenesis of RA by triggering autoimmunity (118). EVs containing major histocompatibility complex class II (MHC II) molecules can be loaded with citrullinated peptide antigens and presented to T cells (119). These peptide antigens can be recognized by autoreactive T cells and trigger the production of anticitrullinated protein antibodies, a key hallmark for RA (120, 121). Autophagy appears to contribute to the generation of citrullinated peptide and EVs in RA (122, 123). It also promotes the citrullinated peptide-MHC II interaction in RA synovial fibroblasts (124). The autophagic system releases cellular content through EVs (122, 125), which can propagate autoantigens and potentially contribute to joint inflammation in RA patients (126) (**Figure 2**).

The serum sEV mRNA profiles in RA patients differed from those of healthy controls and individuals with OA. A combination of differentially expressed mRNAs achieved an AUC of 0.845 in distinguishing RA from OA (92) (**Table 1**).

## 4 EV as a promising therapeutic agent for RA

EVs have gained interest as a potential cell-free therapy due to their low immunogenicity, tumorigenicity, and ease of management (38, 130). They are considered a promising approach for treating RA and may be used as drug delivery vehicles, including as nanocarriers to enhance the therapeutic effect of glucocorticoids in RA treatment (93, 131–133).

Research indicates that EVs from bone marrow macrophages lacking miR-100-5p exacerbate RA progression. In contrast, EVs overexpressing miR-100-5p help reduce inflammation and inhibit the proliferation of RA-FLS in RA (134). EVs from M2 macrophages, loaded with plasmid DNA for IL-10 and betamethasone sodium phosphate, reduced RA inflammation by promoting M1-to-M2 polarization and enhancing anti-inflammatory cytokine secretion (135). IL-4 delivered via small EVs (sEVs) showed a stronger anti-inflammatory effect in mice with CIA than soluble IL-4, indicating greater immunomodulatory potential (136). Additionally, macrophage-derived EVs loaded with IL-10 could be targeted to inflamed areas using noninvasive ultrasound, offering a promising strategy for macrophage polarization to M2 phenotypes in RA treatment (137).

A hybrid nanovesicle (HNV) combining an M1 macrophage membrane with exosome-mimic nanovesicles from M2 macrophages, loaded with black phosphorus sheets, can eliminate inflammatory cells in RA through near-infrared irradiation (138). Apoptotic EVs from macrophages and osteoclasts show synergistic effects in RA joints by reducing synovial inflammation, restoring cartilage, reversing bone erosion, and preserving joint structure (139). Additionally, EVs from immunosuppressive DCs can inhibit the onset and reduce the severity of CIA in mouse models (140). EVs from Indoleamine 2,3-dioxygenase-expressing DCs also demonstrated anti-inflammatory effects in murine models with CIA (141).

EVs engineered to carry super repressor IkB (srIkB), an NF-κB inhibitor, significantly reduced inflammatory cytokine production in PBMCs and synovial fibroblast mononuclear cells (SFMCs) collected from RA patients. Moreover, srIkB EVs treatment showed notable decreases in inflammation, cartilage degradation, and bone erosion in the joint tissues of CIA mice (85).

MSCs are a promising alternative for treating RA due to their immunomodulatory capabilities (142, 143) (**Table 2**). More importantly, the potential of EVs derived from MSCs in immunomodulation and tissue regeneration presents a novel concept for treating rheumatism (5, 144, 145). MSC EVs transfer non-coding RNAs that modulate crucial signaling pathways in the development of RA (34). Different miRNA and lncRNA cargos delivered by MSC-EVs influence RA disorders through the NF-κB and MAPK pathways (51). Moreover, EVs released from MSCs have been identified as important signaling molecules that play a role in the healing process by modulating the local microenvironment with anti-inflammatory properties (146, 147).

**Table 2:** Therapeutic roles of various miRNA, lncRNA, and circRNA EV cargos in RA.

EV cargo	Source cell	Target cell	Target molecule	Function	Ref.
**miR-451a**	UCMSC	RA Synovial Fibroblast	ATF2	• Inhibition of RA-FLS proliferation, migration, and invasion	(152)
**miR150-5p**	BMSC	FLS	MMP4 & VEGF	• Decreased joint damage • Inhibit synovial cell hyperplasia and angiogenesis	(159)
**miR-21**	BMSC	FLS	TET1	• Reduce inflammatory cytokine secretion • Alleviate RA progression	(160)
**circFBXW7 (circ RNA)**	BMSC	FLS	miR-216a-3p	• Inhibited proliferation, migration, and inflammation in RA-FLSs • Inhibit RA damage	(161)
**miR-205-5p**	BMSC	RA-FLS	MDM2	• Suppresses inflammation	(162)
**miR-320a**	BMSC	FLS	CXCL9	• Reduced activation, migration, and invasion of RA-FLS • Reduce severity of arthritis	(163)
**miR-378a-5p**	BMSC	HSMECs	IRF1	• Promotes proliferation, migration and angiogenesis of HSMEC	(148)
**circEDIL3(circ RNA)**	SMSC	FLS	miR-485–3p	• Decreased VEGF expression • Reduced severity of arthritis	(164)
**miR-106b**	Synovial fibroblast	Chondrocytes	PDK4	• Suppression of chondrocyte proliferation and migration • Reduces RA progression	(165)
**miR-433-3p**	SMSC-EV	FLS	FOXO1	• Inhibition of VEGF expression • Reduced severity of arthritis	(166)
**TRAF1-4:1(lncRNA)**	RA-FLS	Chondrocytes	miR-27a-3p	• Inhibit chondrocyte proliferation and migration • Breakdown ECM	(167)
**FGL1**	BMSC	RA-FLS	NA	• Impair RA-FLS viability • Enhance RA-FLS apoptosis	(168)
**miR223**	BMSC	Macrophage	NLRP3	• Suppression of inflammation	(65)
**miR-486-5p**	RA-FLS	Osteoblast	Tob1	• Enhance osteoblast differentiation	(169)
**miR-148a-3p**	GMSC	FLS	IKKB	• Inhibit migration of RA-FLS • Inhibit cartilage degradation	(71)
**miR-140-3p**	UCMSC	FLS	SGK1	• Reduced joint injury	(170)
**miR-124a**	MSC	FLS	NA	• Promote apoptosis of FLS cell • Inhibit proliferation and migration of FLS cell line	(171)

ATF2; activating transcription factor 2, BMDM; bone marrow-derived macrophage, CXCL 9; chemokine ligand 9, circRNA; circular RNA, ECM; extracellular matrix, FGL1; fibrinogen-like protein 1, FLS; Fibroblast-like synoviocytes, FOXO1; forkhead box o1, GMSC; gingival mesenchymal stem cell, HSMECs; human synovial microvascular endothelial cells, IKKB; inhibitor of nuclear factor kappa B kinase, IRF1; Interferon regulatory factor 1, MMPR; matrix metalloproteinase, MDM2; mouse double minute 2, MSC; mesenchymal stem cell, NA; not available, NLRP3; NOD-, LRR- and pyrin domain-containing protein 3, PDK4; pyruvate dehydrogenase kinase 4, RA-FLS; rheumatoid arthritis-fibroblast like synoviocytes, SGK1; serum and glucocorticoid-inducible kinase 1, SMSC; synovial mesenchymal stem cell, TET1; Tet methylcytosine dioxygenase 1, Tob1; Transducer Of ERBB2, 1, TRAF1-4:1; tumor necrosis factor-associated factor 1, UCMSCs; umbilical cord mesenchymal stem cells, VEGF; vascular endothelial growth factor.

The bold text indicates Therapeutic biological molecules carried by EVs.

EVs derived from human embryonic stem cells MSCs reduce inflammation, cartilage degradation, and bone loss, primarily through the modulation of M2 macrophages in arthritis mouse models (64). Additionally, miR-378a-5p from BMSC-derived EVs enhances the proliferation, migration, and angiogenesis of human synovial microvascular endothelial cells by suppressing the IRF1/STAT1 pathway, contributing to the prevention of RA (148).

FLS are crucial in the progression of RA, making them a target for potential treatments (149, 150). EVs from human GMSC have been shown to reduce arthritis progression by decreasing the invasiveness of synovial fibroblasts and protecting cartilage, suggesting therapeutic benefits for RA (71, 151). Additionally, EVs from human umbilical cord MSCs containing miR-451a inhibit the proliferation, migration, and invasion of RA synovial fibroblasts, improving arthritis in rat models (152). BMSC-derived EVs elevated miR-34a levels, reducing RA inflammation and inhibiting RA-FLS proliferation by targeting the cyclin I/p53/ataxia-telangiectasia mutated signaling pathway (153).

MSC-derived EVs infused with curcumin effectively regulate the proliferation and inflammatory response of RA-FLS, significantly reducing anti-apoptotic proteins and inflammatory mediators (154). EVs from IFN-β-primed MSCs also inhibit RA-FLS migration and surface marker expression, showing therapeutic potential for RA (82). Additionally, transfected MSC EVs carrying the lncRNA HAND2-AS1 downregulate the pathogenic miR-143-3p, inhibiting RA-FLS proliferation and motility while inducing apoptosis in *in vitro* experiments (155).

A drug delivery system using adipose tissue-derived MSC EVs successfully delivered icariin to joints, reducing arthritis in rats with CIA by shifting macrophage polarization from pro-inflammatory M1 to anti-inflammatory M2 (156). These EVs enhanced therapeutic effectiveness by modulating macrophage diversity, especially when the MSCs were metabolically engineered to modify EV surface properties (157). Engineering modifications also improved the bone-targeting ability of MSC-EVs, reducing systemic side effects and increasing their clinical application potential (158).

## 5 Conclusion and future perspectives

EVs can modulate innate and adaptive immune responses in experimental RA models. They can transfer different noncoding RNA molecules that regulate gene expression of recipient cells. Molecular EV-cargos, including miRNAs, lncRNAs, mRNAs, and differentially expressed proteins, hold great potential as biomarkers for diagnosing RA. Additionally, MSC-EVs containing various types of miRNAs, lncRNAs, and circRNAs suppressed inflammation and the pathogenic activities of FLS in RA. EVs can also serve as carriers for existing medications. In summary, EVs can inhibit RA immunopathogenesis, reduce the disease’s progression, and serve as promising biomarkers for its diagnosis. Nonetheless, additional research including gene enrichment and pathway analysis is required to detect changes in key signaling pathways and immunoregulatory networks in immune cells exposed to EVs. This will help to completely unravel the molecular mechanisms underlying the immunomodulatory effects of EV cargos in RA.
